# Bayesian Optimization–Enhanced Machine Learning for Osteosarcoma Risk Stratification Based on Sphingolipid Metabolism

**DOI:** 10.1155/humu/2904964

**Published:** 2025-07-11

**Authors:** Yujian Zhong, Ruyuan He, Zewen Jiang, Queran Lin, Fei Peng, Wenyi Jin

**Affiliations:** ^1^Department of Orthopedics, Renmin Hospital of Wuhan University, Wuhan, Hubei, China; ^2^Department of Thoracic Surgery, Renmin Hospital of Wuhan University, Wuhan, Hubei, China; ^3^Department of Primary Care and Public Health, School of Public Health, Faculty of Medicine, Imperial College London, London, UK; ^4^Clinical Research Design Division, Clinical Research Center, Breast Tumor Center, Guangdong Provincial Key Laboratory of Malignant Tumor Epigenetics and Gene Regulation, Sun Yat-Sen Memorial Hospital, Sun Yat-Sen University, Guangzhou, Guangdong, China; ^5^Department of Biomedical Sciences, City University of Hong Kong, Hong Kong SAR, China

**Keywords:** Bayesian optimization, Elastic Net, osteosarcoma, sphingolipid metabolism, XGBoost

## Abstract

**Background:** Heterogenized sphingolipid metabolism (SM) drives osteosarcoma tumorigenesis and its tumor-promoting microenvironment. State-of-the-art bioinformatic tools, such as machine learning, are essential for dissecting the prognostic value of SM by investigating its molecular and cellular mechanisms.

**Methods:** A tailored machine learning pipeline was established by integrating Cox regression, 5-fold cross-validation, Elastic Net, eXtreme Gradient Boosting (XGBoost), and Bayesian optimization (for hyperparameters tuning) to foster an SM Elastic Net-XGBoost (SNEX) prognostic model, interpreted by the Shapley additive explanations (SHAP) algorithm. The alterations in molecular pathways and immune microenvironment–driven unfavorable prognosis of SNEX-identified high-risk osteosarcoma were further investigated. The SNEX predicted results have also been clinically and experimentally validated.

**Results:** We identified 22 critical SM prognostic genes for Bayesian-optimized SNEX. This model provided outstanding estimates of the prognoses of osteosarcoma patients (C-index of 1.000). Its robustness was confirmed in the independent test set with a high area under the curve (AUC) of 0.875 at 1 year, 0.930 at 3 years, and 0.930 at 5 years. SNEX also significantly outperformed all previous genetic prognostic signatures with a significantly higher net benefit of decision curves and higher AUCs. ACTA2 was the most pivotal gene critical to the negative prediction of SNEX, while BNIP3 was for positive prediction. Mechanistically, SNEX-identified high-risk osteosarcoma suffered unfavorable prognoses due to dysregulation of many critical metabolic/inflammatory/immune biologic processes and immunosuppressive microenvironment, with reduced infiltration of 14 types of immune cells (macrophages, CD8+ T cells, NK cells, etc.). Notably, SNEX highlighted TERT as the most remarkable SM prognostic gene. Clinical osteosarcomas with high expression of TERT exhibited more significant malignant characteristics than others, as evidenced by their higher proliferation efficiency. In addition, all the experiments in vitro and in vivo validated that inhibiting TERT abundance reduces the proliferation, invasion, and migration capabilities of osteosarcoma cells.

**Conclusions:** This study is a first-hand report employing a tailored machine-learning pipeline for dissecting the prognostic value and roles of SM in osteosarcoma. The present study fostered a SNEX for risk-stratification with outstanding accuracy and offered deep insights into SM-mediated pathways and microenvironment dysregulation in osteosarcoma.

## 1. Introduction

Osteosarcoma (OS) is one of the most frequent idiopathic malignant bone sarcomas in children and adolescents, with poor prognosis and high metastasis rates [1, 2]. Current treatment for OS involves neoadjuvant chemotherapy and surgical resection, which has increased the 5-year survival rate for affected individuals having confined OS to 60%–70%. In the last 35 years, the 5-year survival rate for patients suffering from metastatic, recurrent, or surgically inoperable OS has remained unsatisfactory below 20% [3–6]. The unsatisfied prognosis of OS is characterized by a high degree of tumor heterogeneity fueled by a high genetic instability [7]. Therefore, it is imperative to find reliable novel biomarkers to aid patient risk assessment and develop individualized treatment and nursing regimens.

Sphingolipids are bioactive chemicals affecting cancer cell signaling, thereby promoting tumor suppression and patient survival. Changes in sphingolipid metabolism (SM) fuel cancer development and can be a potential target for developing novel chemotherapeutic drugs [8]. Bioactive sphingolipid metabolites potentially serve as important biomarkers for cancer and aid in monitoring disease progression and direct therapy [9]. Significant progress has been made in understanding how SM and related signaling govern these processes in response to anticancer treatments. Sphingolipids critically regulate the cell death mechanisms of the tumor cells [10]. Recent research advances have used innovative molecular, genetic, and pharmacological approaches to target sphingolipid signaling pathways in tumor cells. Such approaches have helped highlight sphingolipids' role and downstream targets in regulating tumor formation and response to chemotherapy, radiation, and/or immunotherapy [11, 12]. Numerous cancer therapeutic research have studied the role of SM in various cancers. Nonetheless, the function of OS genes engaged in SM is not well characterized.

Machine learning, a novel type of artificial intelligence, is increasingly becoming prominent in analyzing medical data and exhibited the potential to promote treatment strategies [13–15]. Machine learning automatically learns from input data and reliably predicts output values based on identifying patterns and trends in the data [16]. eXtreme Gradient Boosting (XGBoost) is a large-scale machine-learning approach initially described in 2016 [17]. It is more advantageous than the gradient-boosting decision tree (GBDT). A single decision tree is a simple and inefficient classifier. However, a tree ensemble model, such as the random forest and GBDT, may be much more accurate, and iteratively developing XGBoost might reduce function loss. XGBoost, in contrast to GBDT, adopts the random forest method of “feature subsampling” to prevent overfitting. The XGBoost algorithm has been used widely in the corporate sector but rarely in the medical research field [18].

In the present study, we dissected the impacts of SM on the prognoses of OS by using a tailored machine learning pipeline to develop an SM Elastic Net–XGBoost (SNEX) for OS risk stratification. The SNEX contained four steps, including feature selection, training, performance testing, and interpretation. Then, the underlying mechanisms leading to the unfavorable prognoses of SNEX-identified high-risk OS were further investigated.

## 2. Materials and Methods

### 2.1. Patients

The gene expression patterns of frozen tumor tissue specimens from the Therapeutically Applicable Research to Generate Effective Treatments (TARGET) project were retrospectively evaluated in this study. Patients without survival information were eliminated from this investigation, and 85 patients from the TARGET cohort were included for further analysis. Patients with OS were randomly allocated in an 8:2 ratio to training and test sets using the train_test_split function from the scikit-learn package (Version 0.24.2) in Python (Version 3.8.1). The randomization was performed without stratification by clinical variables, and a fixed random seed was applied to ensure reproducibility. This 80/20 split is a commonly adopted practice in machine learning to balance training model depth and validation robustness.

Furthermore, we collected six OS samples that derive from Renmin Hospital of Wuhan University. Written informed consent was obtained from all participants, and ethical approval was granted by the Ethics Committee of Renmin Hospital of Wuhan University (Approval Number: 2017K-C015).

### 2.2. SNEX Pipeline

Feature selection was a two-step workflow. The first step identified the prognostic features, which exhibited significant impacts on prognosis of OS patients, by using univariable Cox regression from the lifelines package in Python. The features with *p* value < 0.01 were held as prognostic features. The second step was aimed at removing prognostic features with less impacts on prognosis of OS patients and collinearity. In the second step, we used Elastic Net, of which the hypermeters were tuned by Grid Search and 5-fold cross-validation, to further select prognostic features. Lifelines, scikit-learn, and scikit-survival packages were used to conduct these methods in Python.

XGBoost was trained with Bayesian optimization with optuna and xgboost packages in Python. For Bayesian optimization, Tree-Structured Parzen Estimator (TPE) algorithm was employed for raising 100 trials for hyperparameter optimization.

### 2.3. Validation

The prognostic capability of SNEX was validated through cross-validation between the training and test datasets. Prognostic accuracy was assessed using the area under the curve (AUC) derived from the time-dependent receiver operating characteristic (TROC) analysis and the concordance index (C-index). Patients within each dataset were classified into low-risk or high-risk overall survival groups based on the optimal cutoff threshold identified via TROC analysis in the training set. Survival data were evaluated by the Kaplan–Meier curves, employing the product-limit estimation method as previously described [18].

The optimal cutoff threshold was selected based on the Youden Index, following methods reported in earlier literature [18]. Specifically, in the training set, the Youden Index was calculated for each point along the TROC curve, with the point yielding the highest Youden Index chosen as the threshold. The Kaplan–Meier and TROC curves were generated using the rms, survival, survivalROC, and survminer packages in R.

### 2.4. Interpretability

SHAP (Shapley additive explanations) is a method grounded in game theory designed to interpret the outputs of various machine learning models. It integrates optimal credit assignment and local interpretability by leveraging traditional Shapley values from game theory, along with their associated extensions [19]. In this study, we applied the SHAP framework using the SHAP Python library to interpret the results derived from SNEX.

### 2.5. Underlying Mechanism Leading to Survival Difference

We conducted Gene Ontology (GO) annotation analyses to understand the biological functions of SNEX-associated genes. Gene Set Enrichment Analysis (GSEA, Version 4.0.3) was utilized to identify gene sets that were significantly enriched or depleted between the low-risk and high-risk OS. The reference list of genes was based on a gene set from the KEGG that was provided by the GSEA [20].

### 2.6. General Microenvironment and Immune Microenvironment Difference Between SNEX-Identified High- and Low-Risk OS

Stromal and immune cell infiltration in malignant tissues was quantified with the ESTIMATE algorithm, which assesses the infiltration levels of cancerous and noncancerous cells based on distinct transcriptional signatures of tumor cells [21, 22]. This algorithm's reliability has been confirmed across multiple cancer types. In this study, we implemented the ESTIMATE method via the ESTIMATE package in R to quantify cellular infiltration within the tumor microenvironment. Specifically, stromal scores, immune scores, ESTIMATE scores, and tumor purity were calculated to represent the infiltration extents of stromal cells, immune cells, normal cells, and tumor cells, respectively.

We further analyzed the infiltration of various immune cells through a single-sample Gene Sets Enrichment Analysis (ssGSEA), which independently measured 29 cellular immune components in each of the clinical samples of OS. The ssGSEA comprehensively analyzes immune cell infiltration in low- and high-risk OS samples. This method was described in our previous work [23]. ssGSEA was performed on the transcriptome data of each OS sample with the GSVA and GSEABase packages in R. The gene sets used in this program correspond to the published gene sets of 29 cellular immune components [23]. The final atlas comprised the dysregulated immune cellular components in OS, whose enrichment scores were scaled using the pheatmap packages.

### 2.7. Immunofluorescence

Immunofluorescence assay was conducted according to the previous study [23]. The primary antibodies were ki67 (Abcam, Cat. No. ab16667) and TERT (Abcam, Cat. No. ab230527). The secondary antibodies were Alexa Fluor 488-conjugated secondary antibody (Servicebio, Cat. No. GB25303) and Cy5-conjugated secondary antibody (Servicebio, Cat. No. GB27303).

### 2.8. Cell Line and Culture Conditions

To evaluate the in vitro role of TERT in OS, we acquired the OS cell lines 143b and U2OS from Procell (Wuhan, China) for experimental analysis. The cells were maintained in high-glucose DMEM containing 10% fetal bovine serum (FBS) and 1% penicillin/streptomycin (Pen/Strep) and incubated at 37°C with 5% CO₂ in a humidified environment. Cells were passaged every 2–3 days by using 0.25% trypsin for detachment. Experiments exclusively utilized cells in their logarithmic growth phase to ensure consistency and reproducibility.

### 2.9. Cell Transfection

For TERT gene silencing, siRNA specific to TERT was introduced into 143b and U2OS cells using Lipofectamine 3000 (Thermo Fisher). Cells were plated at a density of 1 × 10^5^ cells per well in 6-well plates and incubated overnight to facilitate adherence. On the next day, 20 nM siRNA was combined with Lipofectamine reagent following the manufacturer's protocol and added to the cells for 48-h incubation. The effectiveness of gene silencing was confirmed by quantitative reverse transcription PCR (qRT-PCR) and Western blot analyses, verifying the decreased expression levels of TERT.

### 2.10. qRT-PCR

Total RNA was isolated from transfected 143b and U2OS cells utilizing the RNeasy Plus Mini Kit (Qiagen) according to the provided guidelines. RNA concentration and integrity were evaluated with a NanoDrop spectrophotometer (Thermo Fisher). Reverse transcription was conducted with 1 *μ*g of RNA using the PrimeScript RT Reagent Kit (Takara), following the manufacturer's instructions to synthesize cDNA.

For qRT-PCR, primers specific to TERT and GAPDH (internal reference) were designed with Primer3 software. Amplification reactions were performed using SYBR Green PCR Master Mix (Thermo Fisher) in a QuantStudio 3 real-time PCR system (Applied Biosystems). The cycling conditions included an initial denaturation step at 95°C for 10 min and then 40 cycles of 95°C for 15 s and 60°C for 1 min. Relative expression levels of TERT were determined by the ΔΔCt method, using GAPDH as the normalization control.

### 2.11. Western Blot Analysis

For analyzing protein expression, cells were lysed in preparation for Western blot analysis. Proteins extracted from transfected 143b and U2OS cells were separated using SDS-PAGE and then transferred onto PVDF membranes. The membranes were blocked in TBST containing 5% skimmed milk for 1 h at room temperature. Subsequently, membranes were probed overnight at 4°C with specific primary antibodies against TERT and GAPDH (internal loading control). Following thorough washing, membranes were incubated with appropriate horseradish peroxidase-linked secondary antibodies for 1 h at room temperature. Immunoreactive bands were detected using enhanced chemiluminescence (ECL), and images were captured using a ECL imaging system.

### 2.12. CCK-8 Assay

For cell viability assessment, 143b and U2OS cells were plated at a density of 1 × 10^4^ cells per well into 96-well plates and incubated overnight for attachment. Following experimental treatments, 10 *μ*L CCK-8 solution was added to each well. Plates were then incubated at 37°C for 1–4 h. Subsequently, absorbance was measured using a microplate reader at 450 nm. Viability was calculated based on the absorbance values, reflecting the percentage relative to untreated control cells.

### 2.13. EdU Assay

For evaluating cell proliferation, 143b and U2OS cells were seeded into 96-well plates at a density of 1 × 10^4^ cells per well and incubated overnight to ensure attachment. After treatments, cells were exposed to 20 *μ*M EdU reagent for 2 h at 37°C. Subsequently, cells were fixed with 4% paraformaldehyde at room temperature for 30 min and permeabilized using 0.5% Triton X-100 for 20 min. Cells were then incubated with the click reaction mixture supplied by the manufacturer. The reaction was conducted according to the provided guidelines. Finally, the cells were analyzed to measure proliferation rates.

### 2.14. Colony Formation Assay

To assess colony formation, 143b and U2OS cells were plated at a low density of 500 cells per well in 6-well plates and incubated overnight to facilitate attachment. Following treatment, cells were cultured in complete medium for 10–14 days, with fresh medium replenished every 3–4 days. Colonies formed were fixed using 4% paraformaldehyde for 30 min at room temperature and then stained with crystal violet solution. The staining allowed visualization and counting of colonies to determine colony formation efficiency. Medium changes occurred regularly at intervals of 3–4 days throughout the experimental period.

### 2.15. Transwell Invasion Assay

Cell invasion was assessed using Transwell chambers with inserts precoated with extracellular matrix components to simulate physiological conditions. 143b and U2OS cells were starved in serum-free media for 24 h before the experiment. Subsequently, 1 × 10^4^ cells in serum-free medium were placed in the upper chamber, while medium containing 10% serum was added to the lower chamber as a chemoattractant. After incubation, cells that had migrated to the underside of the membrane were fixed with paraformaldehyde and stained with crystal violet. Cells were quantified by counting in five randomly selected microscopic fields, and the invasion capability was expressed relative to control cells.

### 2.16. Wound Healing Assay

Cell migration was evaluated using a wound healing assay. 143b and U2OS cells were plated into 6-well plates at a density of 5 × 10^5^ cells per well and cultured until confluent overnight. Upon reaching confluence, a uniform scratch was generated in each well using a sterile 200 *μ*L pipette tip. Subsequently, wells were rinsed gently with PBS to remove detached cells and cultured in serum-free medium to inhibit proliferation. Wound closure was monitored and imaged at 0, 24, and 48 h under a phase-contrast microscope (Olympus). Wound widths were measured, and cell migration rates were expressed as percentages based on comparisons to the initial scratch area at 0 h.

### 2.17. In Vivo Study

Animal experiments adhered to the ethical standards set by Wuhan University Renmin Hospital Animal Center. Male nude mice were obtained from Beijing HuaFukang Experimental Animal Center (Beijing, China) and maintained under specific pathogen-free (SPF) conditions at the Animal Center of Wuhan University Renmin Hospital. Animals were acclimated for a period of 1 week before initiating the experiments.

To evaluate tumorigenicity, 5 × 10^6^ cells (either 143b or U2OS) were resuspended in 100 *μ*L of PBS and injected subcutaneously into the right flank of each mouse. Tumor dimensions (length and width) were measured using calipers every 3 days, and tumor volumes were calculated using the equation *V* = (length × width^2^)/2. At the experiment's conclusion, mice were humanely euthanized, and tumors were harvested for subsequent analyses.

### 2.18. Statistical Analyses and Visualization

Raw data utilized in this research were compiled using Python Version 3.8.1 and R software Version 3.6.1 (The R Project for Statistical Computing). Statistical analysis was performed with R Version 3.6.1. Continuous and categorical data correlations were evaluated using Pearson's and Spearman's correlation coefficients, respectively. Group comparisons were carried out using the Wilcoxon test.

## 3. Results

### 3.1. SNEX Methodology

**Figure 1:**
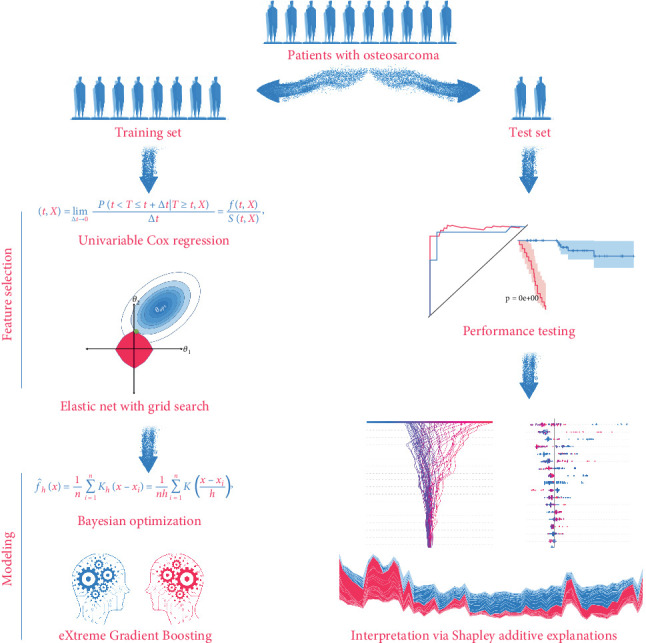
Graphical abstract.

We have depicted the tailored machine learning pipeline to establish SNEX for OS risk stratification in Figure 1. The OS patients were randomly assigned to the training set and test set at a proportion of 8:2. The training set was used for SNEX training and interpretation, and the test set was only for independent testing of SNEX performance.

The feature selection module included two steps. All inputted features were initially filtered by univariable Cox regression to determine the critical prognostic genes for SM. Elastic Net model was then trained by using those prognostic genes for SM and offered us with a features subset by further removing all features with collinearity. Elastic Net model was a penalized Cox model that combined L1 and L2 priors as regularizer; it could remove features with collinearity or fewer impacts on outcome by setting their coefficients as zero. We used 5-fold cross-validation and grid search algorithm to search the best hyperparameter alpha, which determined what features should be removed by Elastic Net model.

The modeling process in this method employed XGBoost and Bayesian optimization. We used 20% samples from the training set as a validation set, which was used to surveil metric change during hyperparameters tuning and XGBoost echoes. TPE algorithm, a Bayesian optimization method, was employed for tuning the hyperparameters. After determining the hyperparameters' search space, TPE was used to construct Gaussian Mixture Models sequentially to approximate the performance of the hyperparameters based on historical measurements. The evaluation metric is Cox negative log-likelihood; the search space is as follows: max_depth (6–10), eta (1e-8–1.0), gamma (1e-8–1.0), and grow_policy (depthwise or lossguide). Subsequently, new hyperparameters were selected to test this model [24, 25]. XGBoost was used as a tool for massively parallel boosting trees. In each iteration, it first identified the first-order and second-order derivative of the loss function, followed by performing the second-order Taylor expansion upon the objective function. Then, it optimized the objective function for approximating function value at each step and finally generated an overall model based on the additive algorithm [26].

The performance of established SNEX was independently tested in the test set. AUC of the ROC curve was employed to evaluate its discrimination and the Kaplan–Meier curve for its accuracy in risk stratification. In the benchmark comparison, AUC of ROC curves and decision curve analyses were performed to verify SNEX's advantages compared to other genetic prognostic signatures.

Although SNEX achieves high predictive accuracy, its machine learning core renders the model a “black box,” potentially eroding clinician trust and slowing clinical adoption; to overcome this limitation, we integrated SHAP directly into the SNEX pipeline. SHAP is a model-agnostic, game-theoretic framework that assigns a Shapley value to every input variable, quantifying how much that variable, alone and in combination with others, pushes the prediction away from a baseline expectation. Its built-in architecture first samples coalitions of features from a background dataset, then applies algorithms optimized for specific model classes to estimate these values, and finally aggregates the local attributions into global importance rankings and intuitive visual summaries. By passing each SNEX prediction through this workflow, we obtain patient-level explanations that show precisely how individual features influence the output, thereby converting the original opaque model into a transparent, trustworthy tool that is better positioned for real-world clinical translation.

### 3.2. Feature Selection and SNEX Training

**Figure 2:**
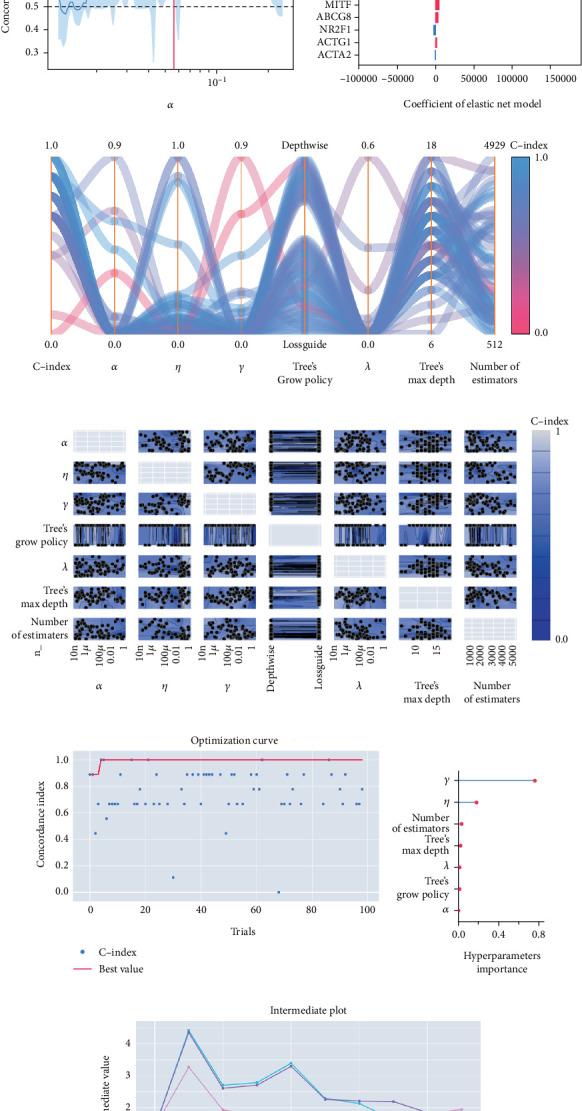
Feature selection and SNEX (Sphingolipid Metabolism Elastic Net-XGBoost) modeling. (a, b) Results of Elastic Net (a regularized Cox regression model). (c, d) History of hyperparameters tuning of SNEX. (e) Optimization curve of SNEX. (f) Hyperparameters' importance of SNEX. (g) Intermediate plot.

The univariable Cox regression identified 28 critical SM genes affecting the prognoses of OS patients (Table 1). These prognostic SM genes were then fed into the Elastic Net model for secondary feature selection. Elastic Net employed C-index as a metric to optimize hyperparameter alpha via Grid Search algorithm with 5-fold cross-validation. The hyperparameter tuning process finally distinguished 0.000391 as the best alpha, subsequently employed to train the best Elastic Net model. The model trained with this alpha achieved the highest C-index of 0.789 ± 0.097 (Figure 2a) and further extracted 22 prognostic SM genes with nonzero coefficients for SNEX modeling (Figure 2b).

Afterward, we launched 100 trials with the TPE algorithm for hyperparameter tuning and employed C-index as a metric and negative log-likelihood as a loss function to train SNEX. The history of hyperparameter tuning, which presented each nonpruned hyperparameter combination and the C-index of the SNEX candidate trained by the corresponding hyperparameter combination, was studied (Figure 2c,d). Trail 4 was the best trail, with the highest C-index and minimal negative log-likelihood to generate SNEX, as demonstrated by the trial's optimization curve (Figure 2e). Therefore, with our tailored training module, the best hyperparameter combination was lambda of 0.000125, several estimators of 1022, tree's max depth of 14, eta of 0.848, gamma of 0.000146, and tree's growing policy of depth-wise. Gamma was the most critical hyperparameter to improve SNEX performance (importance score of 0.76), followed by eta, several estimators, etc. (Figure 2f). These hyperparameters were intermediated for modeling the final SNEX (Figure 2g).

### 3.3. SNEX Performance

**Figure 3:**
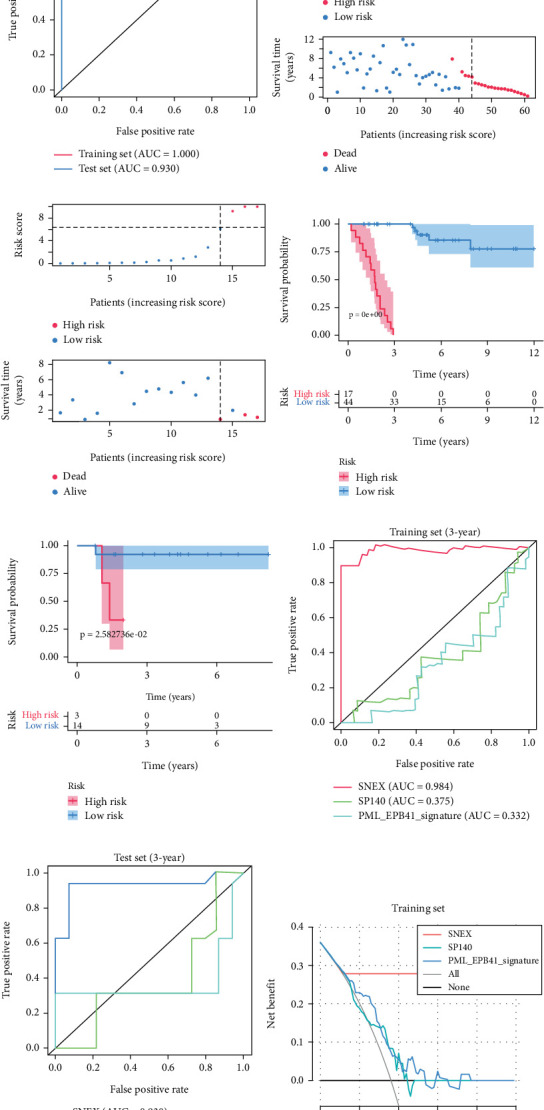
SNEX (sphingolipid metabolism Elastic Net–XGBoost) performance and benchmark testing. (a–c) AUCs (area under the curves) of ROCs (receiver operating characteristic curves) within training and test set. (d, e) SNEX grouping and scoring for osteosarcoma patients in the training and test set. (f, g) Survival analyses of SNEX risk groups. (h–k) Benchmark testing between SNEX and other signatures.

SNEX was an outstanding tool for assisting clinicians in the pre-evaluation prognosis of OS patients based on SM genetic information. As shown in Figures 3, 3, and 3, the AUC values of the ROC curves demonstrated that SNEX achieved strong discrimination performance, particularly in the training set (1.000 at 1 year, 0.984 at 3 years, and 1.000 at 5 years). While these near-perfect AUCs suggest excellent internal fit, they may partially reflect overfitting due to the limited sample size. The high AUCs observed in the independent test set (0.875 at 1 year, 0.930 at 3 and 5 years) offer a more realistic estimation of the model's generalization ability. Afterward, all patients were stratified into high and low-risk groups based on the optimum cutoff value of 6.38 derived from the ROC curve in the training set (Figure 3). SNEX identified that the high-risk patients had significantly less overall survival probability in the training set (Figure 3) and test set (Figure 3), demonstrating the precision with which SNEX distinguished those with worse prognoses.

Furthermore, we tested SNEX performance within benchmark comparison and found it superior to the previous genetic prognostic signature, such as the PML-EPB41 and SP140 models [27]. AUCs of the ROC curve of SNEX at 3 years were 0.984 in the training set, whereas the SP140 and PML-EPB41 signatures only reached AUCs of 0.375 and 0.332, respectively (Figure 3h). The resultant data in the test set was similar (Figure 3i). Additionally, DCA showed SNEX to hold a much higher net benefit than SP140 and PML-EPB41 signatures in the training set (Figure 3j) and test set (Figure 3k). Our findings collectively highlighted the outstanding performance of SNEX for prognosis prediction and evidenced it could be beneficial for oncologists clinically.

### 3.4. Interpretation of SNEX

**Figure 4:**
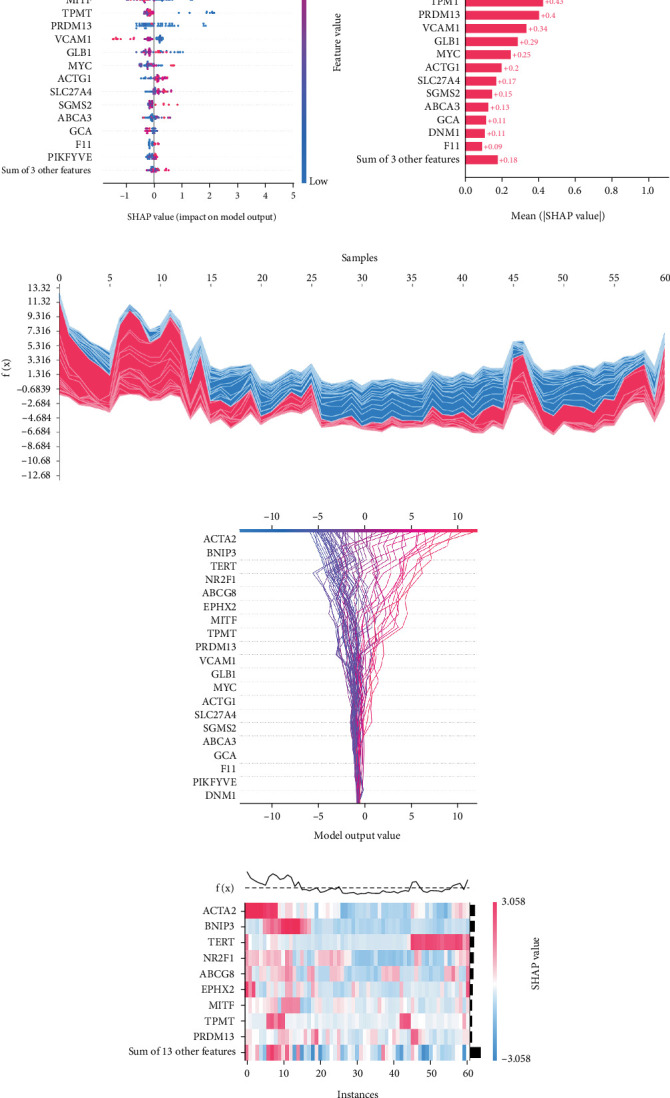
Interpreting SNEX (sphingolipid metabolism Elastic Net–XGBoost). (a) Summary plot generated by SHAP (Shapley additive explanations), illustrating the overall feature impact. (b) Top features ranked by SHAP importance values. (c) Force plot showing the contribution of each feature to individual SNEX predictions. (d) Decision plot visualizing the cumulative contribution of features in the decision-making process for each patient. (e) Contribution of the top-ranking features to SNEX predictions across the cohort.

**Figure 5:**
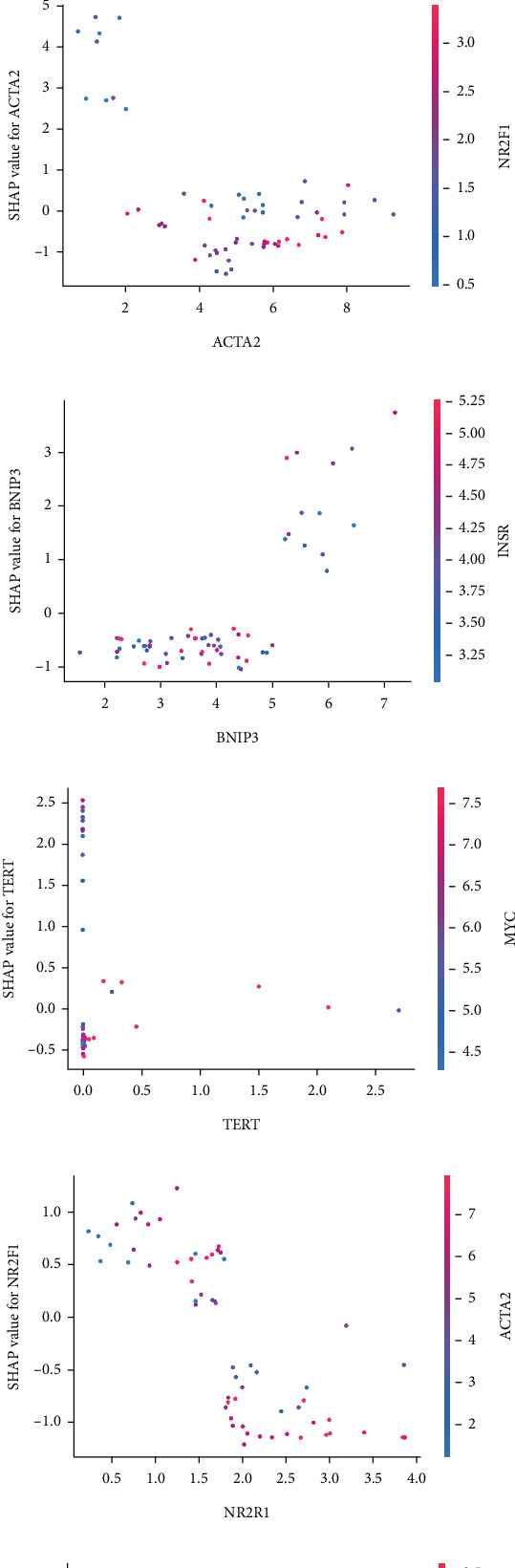
Interpretation of SNEX (sphingolipid metabolism Elastic Net–XGBoost). (a, b) An illustrative example demonstrating the detailed decision-making process of SNEX using SHAP (Shapley additive explanations). (c–g) Dependence plots showing the effects of the top-ranked features on SNEX prediction scores across patients.

The SHAP algorithm enabled us to take a deep insight into the decision-making process of SNEX to improve its clinical translational potential and user trust.  Figure 4a summarizes the contributions of top features to the SNEX prediction of each individual, and ACTA2 was the most critical feature in altering SNEX prediction. Its mean |SHAP value| was 1.01, followed by BNIP3 (0.93), TERT (0.84), etc. (Figure 4b). We also interpreted each prediction's decision-making process with SHAP. The force plot in Figure 4c exhibited each included feature's contribution to each patient. For every individual, features with negative contributions were highlighted with blue and positive contributions were red. SNEX summed up each contribution to render the final predictions (Figure 4d). The top features' contributions and how SNEX exploited these top features are highlighted in Figure 4e, and we found that the alterations of ACTA2, BNIP3, and TERT expression could dramatically increase or decrease the SNEX prediction. Taking a standalone OS patient as an example, TERT positively contributed to SNEX prediction, while ACTA2 featured a negative contribution (Figure 5a,b). For this patient, the most important feature was TERT. However, from the global perspective, the most important SM gene for OS progression was ACTA2, followed by BNIP3, and TERT. The effects of these top features upon SNEX prediction were further investigated. As resultant data presented in Figures 5c, 5d, 5e, 5f, and 5g, ACTA2, TERT, and NR2F1 featured the remarkable impacts to push SNEX prediction to negative, the Spearman rho to their SHAP values were −0.24, −0.46, and −0.84. The BNIP3 and ABCG8 exhibited positive effects on SHAP predictions.

### 3.5. Dysregulated Pathways Regulated by SNEX-Associated Genes

**Figure 6:**
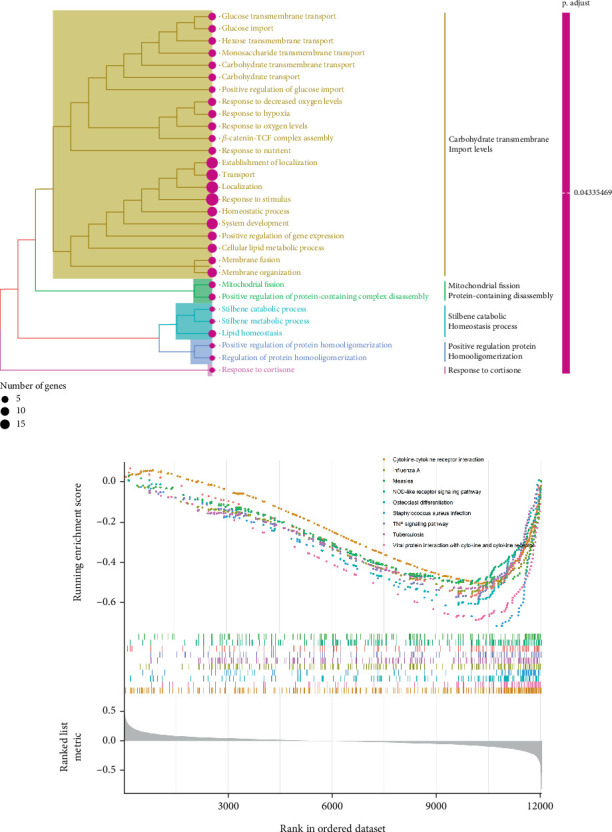
Molecular mechanisms underlying unfavorable prognoses of osteosarcoma patients. (a) Results of Gene Ontology (GO) annotation analysis of SNEX (sphingolipid metabolism Elastic Net–XGBoost)-associated genes. (b) Gene Set Enrichment Analysis (GSEA) identifying dysregulated pathways in high-risk versus low-risk OS groups.

Initially, we performed GO enrichment analysis to identify the biological roles of the SNEX-associated genes (Figure 6a). Most identified genes and activities, including glucose transmembrane import levels and mitochondrial fission protein-containing disassembly, were associated with SM and immunological/inflammatory response. These results suggested that the genes implicated in SNEX primarily govern SM and immune-inflammatory cross-talk-associated pathways, which have long been recognized as the critical biological mechanism underlying tumor growth.

Subsequently, we explored the dysregulated pathways in high-risk OS to uncover any characteristics that led to the difference in survival between low-risk and high-risk OS (Figure 6b). NOD-like receptor signaling pathway and TNF signaling were the most substantially downregulated pathways in high-risk OS, as determined by GSEA using KEGG pathway data. In conclusion, the high-risk OS identified by SNEX exhibited immune-inflammatory crosstalk-associated pathways that were downregulated to prevent immune system disruptions and obtain survival benefits.

### 3.6. Immunosuppressive Microenvironment Underlined the Unfavorable Prognoses of SNEX-Identified High-Risk OS

**Figure 7:**
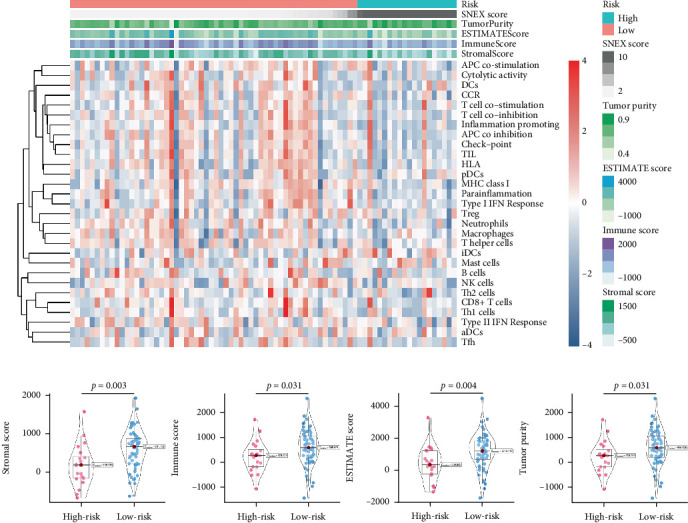
Microenvironment analyses. (a) Global microenvironment and detailed immune microenvironment of high- and low-risk osteosarcoma patients. (b) Differential microenvironment between high- and low-risk osteosarcoma patients.

**Figure 8:**
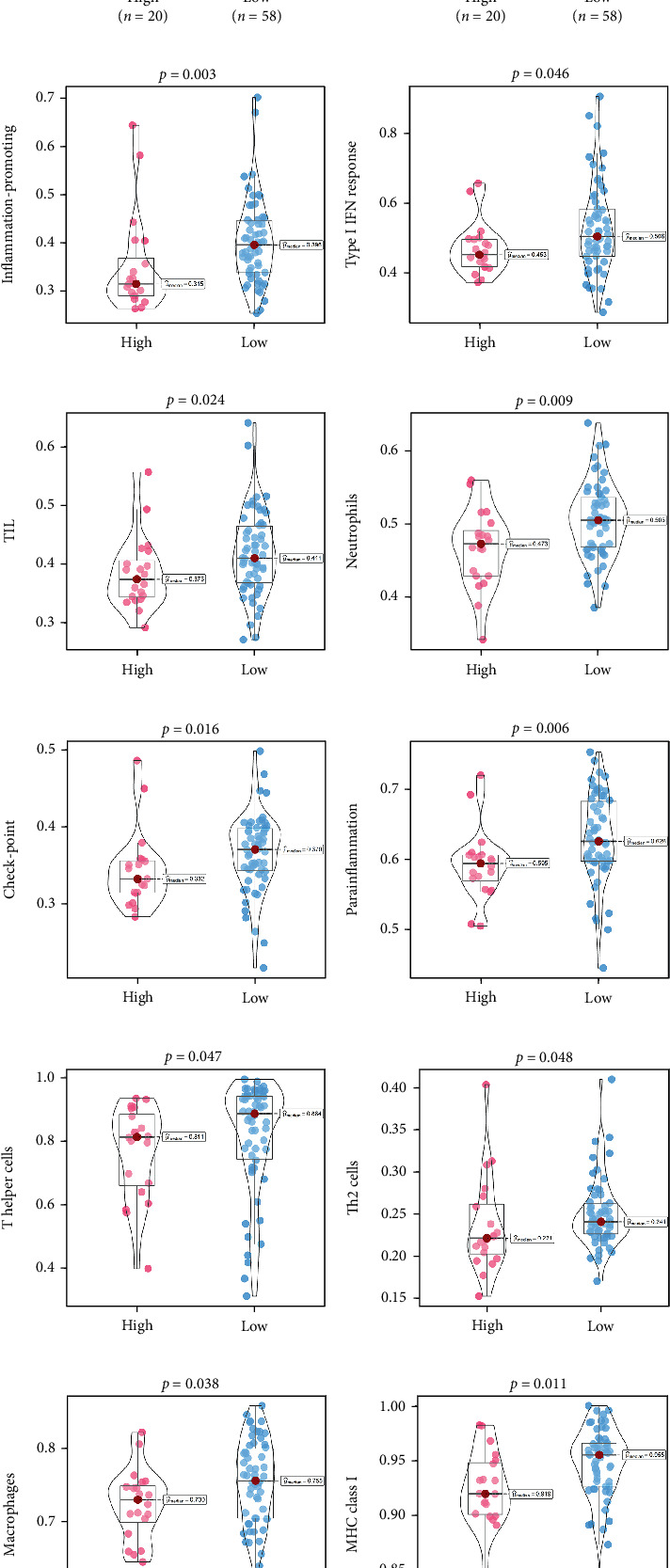
Immunosuppressive microenvironment. (a–n) Differential infiltration of 14 immune cells between high- and low-risk osteosarcoma patients. (o) Correlations between SNEX (sphingolipid metabolism Elastic Net–XGBoost) score and 14 immune cells.

An immune-inflammatory crosstalk-associated pathway dysregulation had been uncovered in our findings above, leading us to profoundly investigate the immune microenvironments of OS, assessed using stromal, ESTIMATE, and tumor purity scores (Figure 7a,b). The SNEX-identified high-risk OS was significantly more likely to have lower stromal (176.18 vs. 586.50), immune (236.12 vs. 599.70), and ESTIMATE (412.30 vs. 1186.20). In contrast, the tumor purity of high-risk samples was significantly higher (77.71% vs. 70.23%). These findings demonstrated that a restricted immune microenvironment fueled the SNEX-identified high-risk OS. Therefore, ssGSEA analysis revealed the specific cellular mechanisms underlying the immunosuppressive microenvironment. As learned from Figure 7, 14 types of cellular immune components have been identified within the high-risk OS. More specifically, the mostly down-infiltrated immune cells was NK cells (log2 fold-change: −0.41, Figure 8a), followed by follicular helper T cells (Tfh, −0.40, Figure 8b), CD8+ T cells (−0.34, Figure 8c), APC coinhibition (−0.21, Figure 8d), inflammation-promoting (−0.19, Figure 8e), Type I interferon (IFN) response (−0.17, Figure 8f), tumor-infiltrating lymphocyte (TIL) (−0.12, Figure 8g), neutrophils (−0.12, Figure 8h), check-point (−0.09, Figure 8i), part inflammation (−0.09, Figure 8j), helper T cells (−0.09, Figure 8k), T helper 2 cells (−0.08, Figure 8l), macrophages (−0.05, Figure 8m), and MHC Class I (−0.03, Figure 8n). Further correlation analyses also revealed that the SNEX scores were significantly negatively associated with the infiltration of these immune cells (Figure 8o). This observation validated that the degree of immunosuppressive microenvironment was increased as SNEX scores had augmented. Such observations confirmed that the specific immune microenvironment fueled the unfavorable prognoses of the high-risk OS.

### 3.7. The Correlations Between SNEX and Clinical Parameters

**Figure 9:**
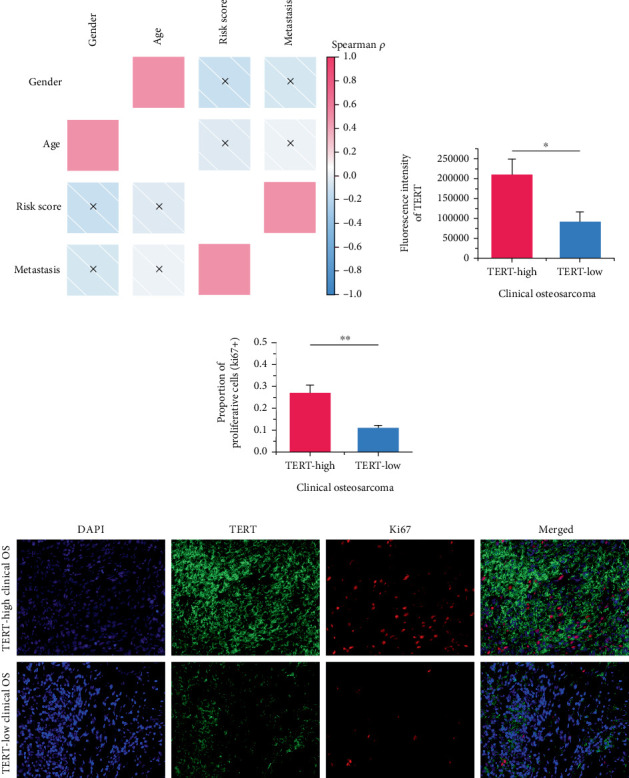
(a) The correlations between SNEX (sphingolipid metabolism Elastic Net–XGBoost) and clinical parameters. (b–d) SNEX-identified risk gene TERT showed higher expression levels in the OS with high proliferation potential.

We performed a clinical correlation analysis to uncover whether the risk score calculated by SNEX is associated with clinical parameters. As results shown in Figure 9a, we found that the risk score was remarkably associated with metastasis, while showing no association with age and gender. These results indicated that the SNEX-identified high-risk patients suffered a much higher probability of metastasis, which is independent of their age and gender.

Moreover, we further validated SNEX in the clinical OS samples. TERT, a risk gene identified by SNEX, was examined to confirm whether its expression was associated with OS progression. Immunofluorescence assay attested that the TERT-high OS exhibited much higher proliferation ability compared to TERT-low OS (Figures 9b, 9c, and 9d), indicating that TERT as a risk factor highly fueled the malignant progression of OS.

### 3.8. Efficient Knockdown of TERT Expression by siRNA

**Figure 10:**
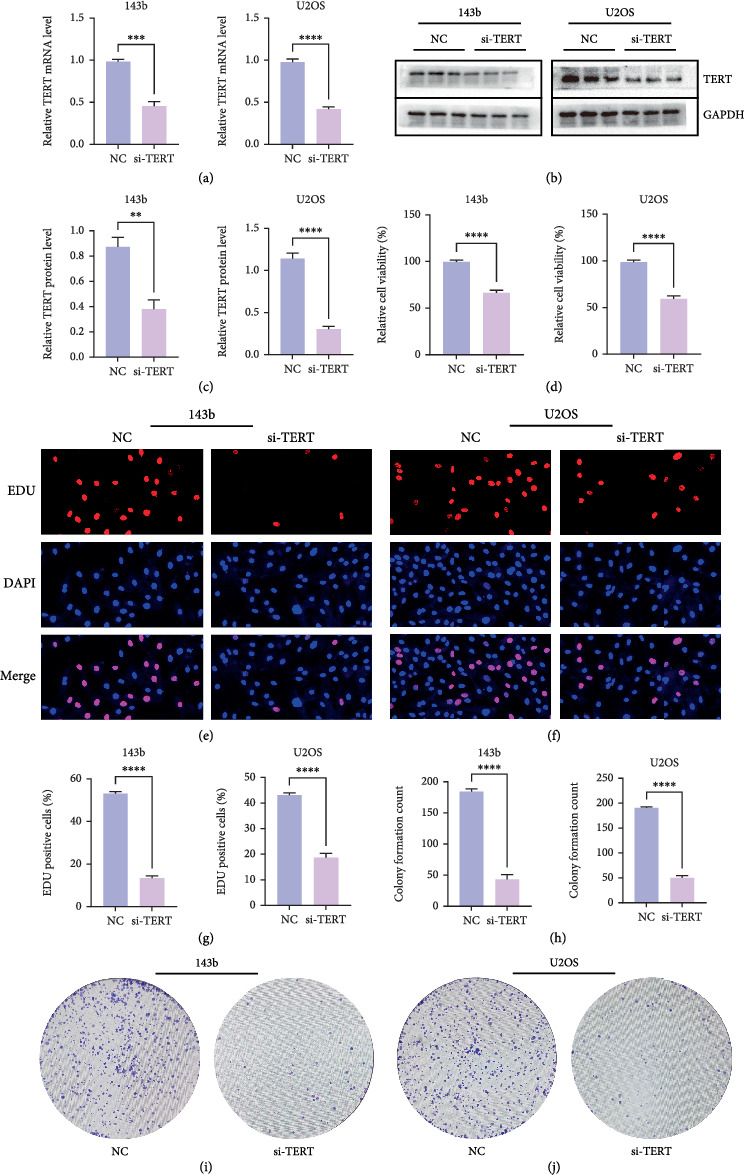
T ERT knockdown inhibits proliferation of 143b and U2OS osteosarcoma cells. (a) qRT-PCR analysis confirming TERT mRNA knockdown in 143b and U2OS cells transfected with si-TERT. (b) Western blot showing TERT protein reduction in 143b and U2OS cells following si-TERT treatment. (c) Quantification of relative TERT protein expression in 143b and U2OS cells. (d) Cell viability analysis showing a significant reduction in si-TERT treated cells compared to NC. (e, f) Representative EDU staining images showing decreased EDU-positive cells in 143b and U2OS cells after TERT knockdown. (g) Quantification of EDU-positive cells indicating reduced cell proliferation in si-TERT groups. (h) Colony formation assays showing a significant decrease in colony numbers in si-TERT treated cells. (i, j) Representative images of colony formation assays in 143b and U2OS cells, illustrating reduced colony growth following TERT knockdown.

The efficiency of TERT knockdown was evaluated by measuring both mRNA and protein levels in 143b and U2OS cells transfected with si-TERT. qRT-PCR analysis revealed a significant decrease in TERT mRNA expression in both cell lines after si-TERT treatment compared to the negative control (NC) (Figure 10a). Western blotting further confirmed the reduction of TERT protein levels in both 143b and U2OS cells (Figure 10b,c). These findings collectively demonstrate the successful knockdown of TERT by siRNA in OS cells.

### 3.9. Knockdown of TERT Inhibits Proliferation of OS Cells

TERT knockdown significantly reduced the proliferation of both 143b and U2OS OS cells. qRT-PCR and Western blotting confirmed efficient TERT silencing at both the mRNA and protein levels. Subsequent assays demonstrated a notable decrease in cell proliferation following TERT knockdown. Cell viability was significantly reduced in both cell lines after si-TERT treatment (Figure 10d). Similarly, the EDU incorporation assay showed a substantial decrease in the percentage of EDU-positive cells in the si-TERT groups compared to the NC (Figures 10e, 10f, and 10g). Colony formation assays also revealed a significant reduction in the number of colonies formed by si-TERT-treated cells, further supporting the inhibitory effect of TERT knockdown on cell proliferation (Figures 10h, 10i, and 10j).

### 3.10. Knockdown of TERT Inhibits Invasion and Migration of OS Cells

**Figure 11:**
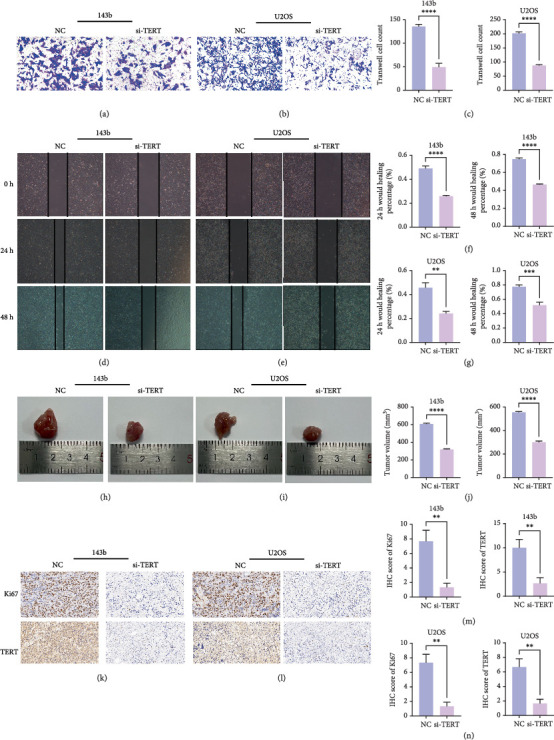
TERT knockdown inhibits migration, invasion, and tumor growth in osteosarcoma cells. a) Transwell invasion assay showing decreased invasion of 143b cells following TERT knockdown. (b) Transwell invasion assay showing decreased invasion of U2OS cells following TERT knockdown. (c) Quantification of the number of invaded cells in 143b and U2OS cells after TERT silencing. (d, e) Wound healing assays showing reduced migration of 143b and U2OS cells after TERT knockdown at 0, 24, and 48 h. (f, g) Quantification of wound healing percentage in 143b and U2OS cells, demonstrating reduced migration in si-TERT-treated groups. (h, i) Representative images of tumor growth in 143b and U2OS xenograft models, showing smaller tumors in si-TERT groups. (j) Tumor volume measurement in 143b and U2OS xenograft models, showing significantly smaller tumors in si-TERT groups. (k, l) Immunohistochemistry (IHC) for Ki67 and TERT expression in 143b and U2OS tumors, indicating reduced proliferation and TERT expression in si-TERT groups. (m, n) Quantification of IHC scores for Ki67 and TERT in 143b and U2OS tumors, showing lower Ki67 and TERT expression in si-TERT groups.

TERT knockdown significantly inhibited the invasion and migration abilities of both 143b and U2OS OS cells. Transwell invasion assays showed a marked reduction in the number of invaded cells in the si-TERT group compared to the NC in both 143b and U2OS cells (Figures 11a, 11b, and 11c). This suggests that TERT silencing suppresses the invasive capacity of OS cells.

Furthermore, wound healing assays demonstrated a significant decrease in the wound closure rate in si-TERT-treated cells compared to the NC group. In 143b cells, the wound healing percentage at both 24 and 48 h was significantly lower in the si-TERT group (Figure 11d,f). Similarly, the healing percentage in U2OS cells was significantly reduced after TERT knockdown, particularly at 48 h (Figure 10e,g). These findings indicate that TERT knockdown impairs the migration ability of OS cells.

### 3.11. TERT Knockdown Suppresses Tumor Growth and Proliferation In Vivo

In vivo experiments demonstrated that TERT knockdown significantly inhibited tumor growth and reduced cell proliferation in both 143b and U2OS OS xenograft models. Tumors derived from si-TERT-treated cells were notably smaller compared to the control group (NC), with a significant reduction in tumor volume observed in both 143b and U2OS cell lines (Figure 11h,i). The tumor volumes were reduced by approximately half in the si-TERT groups compared to the NC groups (Figure 11j), indicating that TERT silencing effectively suppresses tumorigenicity.

Immunohistochemistry (IHC) staining further confirmed the inhibitory effects of TERT knockdown on tumor proliferation. Both Ki67 expression, a marker of cell proliferation, and TERT expression were significantly reduced in the si-TERT tumors (Figures 11k, 11l, 11m, and 11n). These results suggest that TERT silencing not only impairs OS cell proliferation but also effectively limits tumor growth in vivo.

## 4. Discussion

The present study offered a first-hand report created using state-of-the-art bioinformatic tools, that is, machine learning, to dissect the impacts of SM on OS prognosis. The analyses are also aimed at developing a robust prognostication approach for accurate risk stratification in OS. The present findings might help clinicians identify patients with unfavorable prognoses in advance and implement more individualized treatment to prolong overall survival.

Many researchers developed prognostic signatures based on the RNA expression profiling of OS that included immune signature [28], hypoxia signature [29], ferroptosis signature [30], and apoptosis [31]. However, their biological and clinical implications were hindered by certain limitations. The RNA-seq data is a high-dimensional matrix containing numerous features (also known as genes). Such data, therefore, have complicated data point distribution within high-dimensional space. The traditional statistical model used in the previous study is a simple regression model that failed to accurately fit such high-dimensional data due to their complicated data distribution. Therefore, the prognostic signatures based on the statistical model are limited. As we hypothesized, our benchmark comparison firmly demonstrated that the traditional statistical prognostic signatures performed unfavorably compared to our SNEX (Figure 3). Meanwhile, bioinformatics analyses based on these statistical prognostic signatures could only obtain the general tumor chrematistics, the details of which might be ignored, making the performance of these traditional signatures unfavorable. The machine learning model rendered our SNEX high accuracy and robustness. The 3- and 5-year AUCs were all more than 0.9, which potentiated the clinical potential of SNEX.

Sphingolipids originated from a ceramide-constituted common molecular core, and ceramide was also raised from a fatty acid linked to sphingosine [8]. Glycosphingolipids were associated with receptors, adhesion molecules, and signaling players by forming functional clusters upon the plasma membranes to regulate immune cell infiltration and activation [32, 33]. The findings further corroborated the results from previous studies to raise many novel insights into OS progression. The SNEX-associated genes mainly regulated transmembrane transport and immune-inflammatory cross-talking in the high-risk OS, which are strongly associated with unfavorable prognoses of the patients.

The immune microenvironment has long been implicated as the most critical contributor to OS development. TILs have antitumor effects that may serve as an alternative for adjuvant immunotherapy in OS patients who respond poorly to chemotherapy. Lower TIL infiltration is strongly associated with an unfavorable prognosis in OS [34, 35]. Macrophages affect OS progression through macrophage differentiation pathways. M1 macrophages, activated by lipopolysaccharides, T-helper cell 1, and other cytokines, play a critical role in the immune-mediated destruction of OS [36]. Neutrophils also exhibit antitumor cellular activity as they inhibit the proliferation and migration of OS cells [37], whereas T-helper cells reduce OS activity and improve prognosis [38]. The infiltration of these critical immune cells showed a significant decrease in the SNEX-identified high-risk OS. The immunosuppressive microenvironment strongly potentiated the unfavorable prognosis of the high-risk OS.

In addition to the bioinformatics analyses, the in vitro validation of our findings was conducted to further substantiate the clinical relevance of SNEX in OS prognosis. The results from the Transwell and wound healing assays demonstrated that TERT knockdown significantly inhibited both the migration and invasion of 143b and U2OS OS cells. These assays confirmed that the molecular insights predicted by the SNEX model had direct biological implications. Notably, the reduced invasive and migratory capacity of the si-TERT-treated cells aligns with the immune microenvironment alterations suggested by our bioinformatic predictions. The decreased expression of key proliferative markers such as Ki67 and TERT in the tumor tissues further corroborates the relationship between SNEX-associated genes and immune cell infiltration. These in vitro findings not only validate the robustness of SNEX in identifying high-risk OS but also highlight its potential as a promising prognostic tool for guiding therapeutic strategies aimed at improving patient outcomes.

While our model demonstrates robust predictive performance based on SM-related gene expression, we acknowledge that OS is a highly heterogeneous disease influenced by multilayered regulatory mechanisms. These include not only genetic variation but also epigenetic regulation, posttranscriptional and posttranslational modifications, and microenvironmental cues. Our SNEX model captures transcriptomic patterns associated with SM dysregulation, but it does not fully account for upstream genomic drivers (such as TP53 or RB1 mutations) or nongenomic contributors to metabolic rewiring and therapeutic resistance. Future studies integrating multiomics data, including mutational, epigenetic, proteomic, and microenvironmental layers, are warranted to build more comprehensive predictive frameworks and refine the biological interpretation of SM-driven stratification.

The present study still has several limitations that should be acknowledged. First, its retrospective design may introduce potential selection bias and limits the ability to draw causal inferences. Second, the relatively small sample size, especially in the validation cohort, may reduce statistical power and restrict the generalizability of our findings. Therefore, further validation in large-scale, prospective, and multicenter cohorts is warranted to confirm the robustness and clinical utility of SNEX. In addition, the TARGET cohort lacked histological grade information, preventing us from analyzing the potential association between tumor grade and immune cell infiltration. Future studies incorporating complete clinical annotations will be essential to explore these relationships more thoroughly.

## Figures and Tables

**Table 1:** Results of univariable Cox regression.

**Covariate**	**coef**	**exp (coef)**	**se (coef)**	**exp (coef)** **lower** **95%**	**exp (coef)** **upper** **95%**	**z**	**p**
ACTA2	−0.37919	0.684415	0.105942	0.556086	0.84236	−3.57923	0.000345
INSR	0.676647	1.967271	0.234553	1.24226	3.115414	2.884839	0.003916
GLB1	−0.81563	0.442361	0.278435	0.256314	0.763451	−2.92934	0.003397
TERT	1.124358	3.07824	0.306487	1.688186	5.612865	3.668535	0.000244
EPHX2	1.198344	3.314622	0.357911	1.643536	6.684806	3.348156	0.000814
ABCG8	6.173592	479.9071	1.694358	17.33471	13286.1	3.643617	0.000269
TNFRSF1A	−0.71498	0.489203	0.256715	0.295782	0.809106	−2.7851	0.005351
MYC	0.690212	1.994139	0.200347	1.346542	2.953185	3.44509	0.000571
VCAM1	−0.36214	0.696188	0.140488	0.528619	0.916876	−2.57769	0.009946
SGMS2	0.453827	1.574326	0.168781	1.130911	2.191599	2.688858	0.00717
ADCY10	5.930186	376.2246	1.944797	8.318277	17016.14	3.049258	0.002294
DNM1	−0.51147	0.599611	0.191637	0.411859	0.872953	−2.66898	0.007608
ACTG1	−1.00277	0.36686	0.386008	0.172159	0.781757	−2.5978	0.009382
PIKFYVE	0.80437	2.235288	0.256204	1.352859	3.693302	3.13957	0.001692
SLC27A4	−1.0924	0.335411	0.363897	0.164372	0.684427	−3.00194	0.002683
NR2F1	−0.98926	0.371853	0.302799	0.205413	0.673154	−3.26704	0.001087
F11	20.82544	1.11E+09	8.082595	146.0346	8.4E+15	2.576578	0.009978
BNIP3	0.459943	1.583984	0.147821	1.185566	2.116293	3.111494	0.001861
MITF	−0.78099	0.457953	0.299842	0.254445	0.824227	−2.60467	0.009196
STT3A	0.83287	2.299909	0.257685	1.387935	3.811118	3.232127	0.001229
PXN	−0.88752	0.411674	0.309689	0.22436	0.755372	−2.86586	0.004159
CTNNBIP1	−1.23604	0.290533	0.344793	0.147811	0.571063	−3.58487	0.000337
TPD52	0.608971	1.838539	0.187619	1.272833	2.655672	3.245787	0.001171
GCA	−0.98857	0.37211	0.357666	0.184597	0.750096	−2.76394	0.005711
TPMT	−0.80652	0.446411	0.308503	0.243857	0.817209	−2.61428	0.008941
PRDM13	1.516765	4.557459	0.461417	1.844861	11.25854	3.287187	0.001012
ABCA3	0.676715	1.967404	0.21637	1.287415	3.006549	3.127576	0.001763
TUBB6	−0.78383	0.456653	0.296502	0.25539	0.816524	−2.6436	0.008203

Abbreviations: coef, coefficient; *p*, *p* value; se, standard error.

## Data Availability

Data sharing is not applicable to this article as no new data were created or analyzed in this study.
